# Comparison of the Efficacy and Safety of Different Doses of Linaclotide for Patients with Chronic Constipation: A Meta-Analysis and Bayesian Analysis

**DOI:** 10.1155/2021/9923879

**Published:** 2021-10-14

**Authors:** Jiao Yang, YanChang Lei

**Affiliations:** Department of Gastroenterology, WenChang Road 8, Liuzhou People's Hospital, Liuzhou 545000, Guangxi, China

## Abstract

**Background:**

It is ambiguous whether a higher dose of linaclotide provides higher efficacy for chronic constipation (CC) patients. The meta-analysis aimed to assess the efficacy and safety of linaclotide doses ranging from 62.5 *μ*g to 600 *μ*g for CC patients.

**Methods:**

A comprehensive search was conducted, and STATA16 software was used for data analysis.

**Results:**

Seven studies with 4,107 patients were eligible. A significantly enhanced number of completely spontaneous bowel movement (CSBM) responders were found in the extremely low-dose group (OR: 2.94; 95% CI: 1.98–4.34; *p* < 0.001), the low-dose group (OR: 3.24; 95% CI: 2.44–4.31; *p* < 0.001), the medium-dose group (OR: 3.08; 95% CI: 1.46–6.50; *p*=0.003), and high-dose group (OR: 4.79; 95% CI: 3.04–7.54; *p* < 0.001). Bayesian analysis showed the high-dose group obtained the maximum CSBM responder rate (OR: 4.94; 95% credible interval (CrI): 3.22–7.79; probability rank = 0.87) indirectly compared with extremely low-dose, low-dose, and medium-dose groups. However, no significant difference presented in the CSBM responder rate by pairwise comparisons of the different dose groups. Additionally, no more any adverse events occurred in the higher linaclotide dose group (RR: 0.91; 95% CrI: 0.60–1.38) indirectly compared with other dose groups.

**Conclusions:**

High dose of linaclotide could be more effective and safer for CC patients, which need more trials to confirm in the future.

## 1. Introduction

Chronic constipation (CC) may be primary (idiopathic or functional) or secondary to several disorders or medications. CC is characterized by various bowel symptoms, including infrequent bowel movement, hard stools, excessive straining to defecate, a sense of anorectal blockage, anal digitation, and a sense of incomplete evacuation. The most widely used diagnostic criteria of CC are the Rome criteria, and the latest is Rome IV, created in 2016 [1], which distinguishes functional constipation from irritable bowel syndrome with constipation (IBS-C).

CC negatively affects the quality of life, comparable with chronic diseases, such as chronic obstructive pulmonary disease, diabetes, and depression [2, 3]. In contrast, poor quality of life can aggravate CC symptoms. The estimated incidence of CC ranges from 11% to 20% [4–6]. Approximately one in five people presented with CC symptoms will seek medical help [7]. There has been a steady and significant increase in the proportion of ambulatory care related to this disorder [8], and it poses a heavy economic burden for healthcare systems [9, 10]. Thus far, the risk factors for CC identified are advanced age, female, low socioeconomic status, low parental education rates, decreased physical activity, certain medications, stressful life events, physical and sexual abuse, and depression [11, 12].

There are many choices available for the treatment of CC, including changes in defecation habits, increase in fiber intake, and several drugs, such as laxatives and systemically active agents. However, up to 50% of patients report that they are not completely satisfied with current treatment due to inefficient relief from constipation, the side effects of drugs (e.g., bloating and abdominal pain), the lack of predictability of laxative action, and partial improvement of the quality of life [13].

Linaclotide, a peptide homolog of *Escherichia coli* ST toxin, interacts with the guanylate cyclase C (GC-C) located in the enterocyte apical membrane. Activation of the GC-C receptor results in cyclic GMP production that induces the secretion of fluids and electrolytes into the lumen, accelerates colonic transit, and relieves constipation [14]. Many studies have confirmed that linaclotide is superior to the placebo and safe for use in a clinical setting [15–17]. Nevertheless, the optimal dose of linaclotide is still not confirmed. Linaclotide at doses of 145 *μ*g and 290 *μ*g has demonstrated an adequate level of safety and efficacy in two large RCTs conducted on CC patients [18]. However, only the 145 *μ*g dose was approved for CC treatment in adults. A linaclotide dosage of 290 *μ*g once per day is considered safe and effective for IBC-C patients in the USA [19–21]. However, a study from Japan showed that 500 *μ*g linaclotide resulted in a higher monthly responder rate in a global assessment of relief from IBS symptoms, responder rate of completely spontaneous bowel movement (CSBM), and responder rate of abdominal pain or discomfort relief than those with placebo [22], which indicates that a linaclotide dose of 500 *μ*g may be appropriate for IBS-C patients. Similarly, there is ambiguity on whether a high dose of linaclotide provides higher efficacy for CC patients. Therefore, in this study, we aimed to assess the effect and safety of different doses of linaclotide for the treatment of CC.

## 2. Materials and Methods

This meta-analysis was conducted following PRISMA guidelines. A comprehensive search of the PubMed, the Cochrane Library, and the Embase was performed using the search terms: linaclotide and chronic constipation.

### 2.1. Inclusion Criteria

1. Randomized controlled trial (RCT)
2. Participants had to be diagnosed with CC
3. Participants had to be treated using linaclotide
4. The language had to be English

### 2.2. Exclusion Criteria

1. Participants were diagnosed with IBS-C
2. Incomplete data
3. The publication was a letter, comment, editorial, or case report

### 2.3. Study Selection and Data Extraction

The detailed selection process is summarized in the PRISMA flow diagram. Two authors (Jiao Yang and YanChang Lei) independently identified the full-text manuscripts of these studies based on the inclusion criteria and conducted data extraction for the primary and secondary endpoints. Conflicts were resolved by discussion. The primary endpoint was the number of CSBM responders. The secondary endpoints were the number of spontaneous bowel movement (SBM) responders, the number of responders of the global assessment of relief, the number of responders of abnormal bowel habits improvement, and the number of responders of abdominal symptoms relief. The definition of a CSBM/SBM responder was a patient who had reported at least 3 CSBMs/SBMs and an increase of 1 or more CSBMs/SBMs from the baseline at each evaluation point. Responders for global assessment of relief of CC symptoms, abnormal bowel habits improvement, and abdominal symptoms relief were defined as patients with a score of 1 or 2 at each evaluation point.

Moreover, the baseline characteristics, such as country, number of centers, diagnostic criteria for CC, the total number of patients, the proportion of female patients, dose of linaclotide, duration, and outcomes, of the randomized controlled trials were recorded. The extremely low-dose group of patients received 62.5 *μ*g, 72 *μ*g, or 75 *μ*g of linaclotide. The low-dose group of patients received 145 *μ*g or 150 *μ*g of linaclotide. The medium-dose group of patients received 290 *μ*g or 300 *μ*g, and the high-dose group received 500 *μ*g or 600 *μ*g of linaclotide.

### 2.4. Statistical Analysis

STATA 16 software was used to perform the statistical analyses. All outcomes were dichotomous variables. The risk ratio (RR), odds ratio (OR), and 95% confidence intervals (CI) were calculated. Additionally, heterogeneity was analyzed using *I*^2^ or *p* statistics. If *I*^2^ > 50% or *p* < 0.1, indicating heterogeneity, the random-effects model was used. Otherwise, the fixed-effects model was applied. Publication bias was assessed by funnel plots and Begg's test. The quality of the eligible studies was evaluated by Cochrane's risk of bias tool (RevMan 5.3 software) including random sequence generation, allocation concealment, blinding of participants and personnel, blinding of the outcomes assessment, incomplete outcome data, selective reporting, and other biases. In addition, *R* software (3.6.3) with GEMTC and RJAGS packages was utilized to perform Bayesian analysis to rank the optimal dose of linaclotide [23, 24]. The surface under the cumulative ranking (SUCRA) ranging between 0% and 100% was used to rank the probability of the optima dose of linaclotide [23]. 95% credible interval (CrI) was derived from the 2.5^th^ and 97.5%^th^ percentiles.

### 2.5. Level of Evidence

The Grading of Recommendation Assessment, Development, and Evaluation approach (GRADE) was applied to assess the quality of the evidence associated with the meta-analysis primary outcomes results.

## 3. Results

### 3.1. Study Characteristics

**Figure 1:**
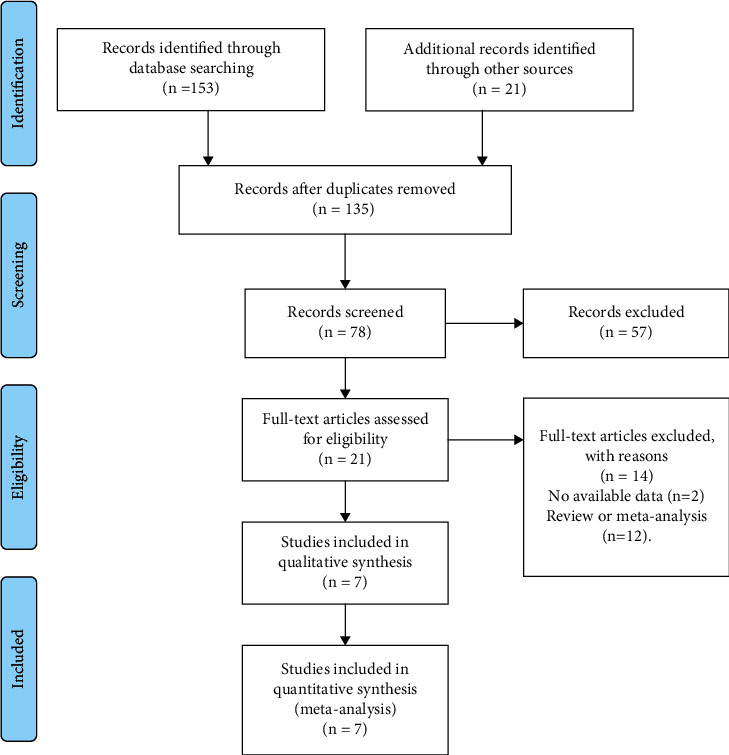
Flow diagram of the literature review process.

Seven studies [18, 25–30] and eight RCTs conducted on 4,107 patients were eligible to be included in the study. Most eligible RCTs were conducted at multiple centers. The CC diagnostic criteria used was Rome II or Rome III. Moreover, the majority of patients were female. The duration of treatment ranged from 2 weeks to 12 weeks and is given in Table 1. The detailed search process is shown in Figure 1.

### 3.2. The Quality of the RCTs

**Figure 2:**
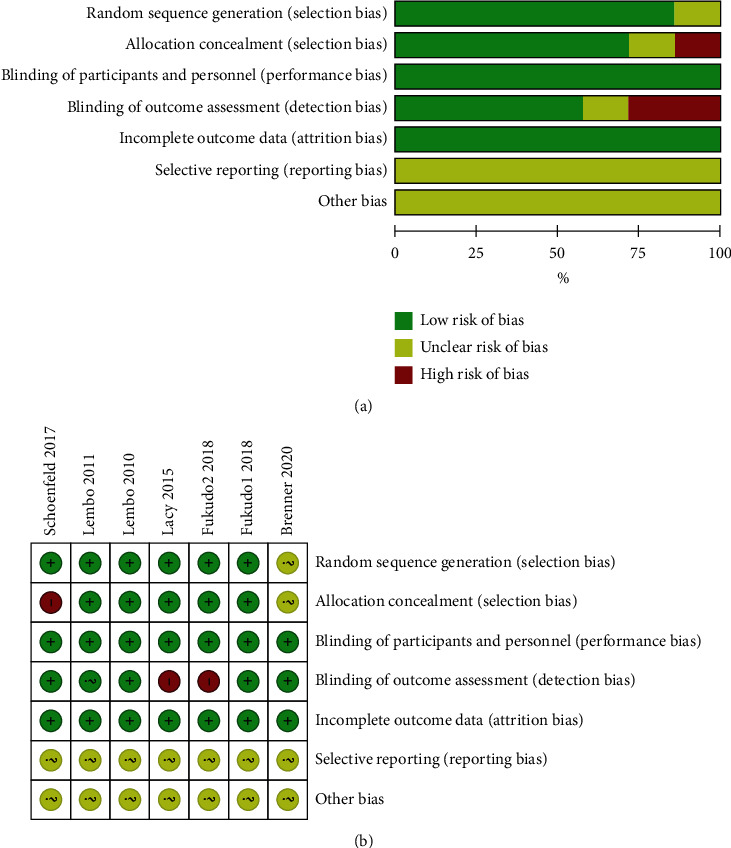
The quality of the RCTs. (a) Risk of bias graph of the eligible studies. (b) Risk of bias summary of the eligible studies.

The quality of all eligible studies was evaluated using the risk of bias tool of the Cochrane collaboration network. Random sequence generation, allocation concealment, blinding of participants and personnel, blinding of outcome assessment, and incomplete outcome data were found to be low risk. Selective reporting and other biases showed an unclear risk of bias, as shown in Figure 2. In total, the quality of these studies was found to be optimal.

### 3.3. Outcomes

#### 3.3.1. CSBM Responders

**Figure 3:**
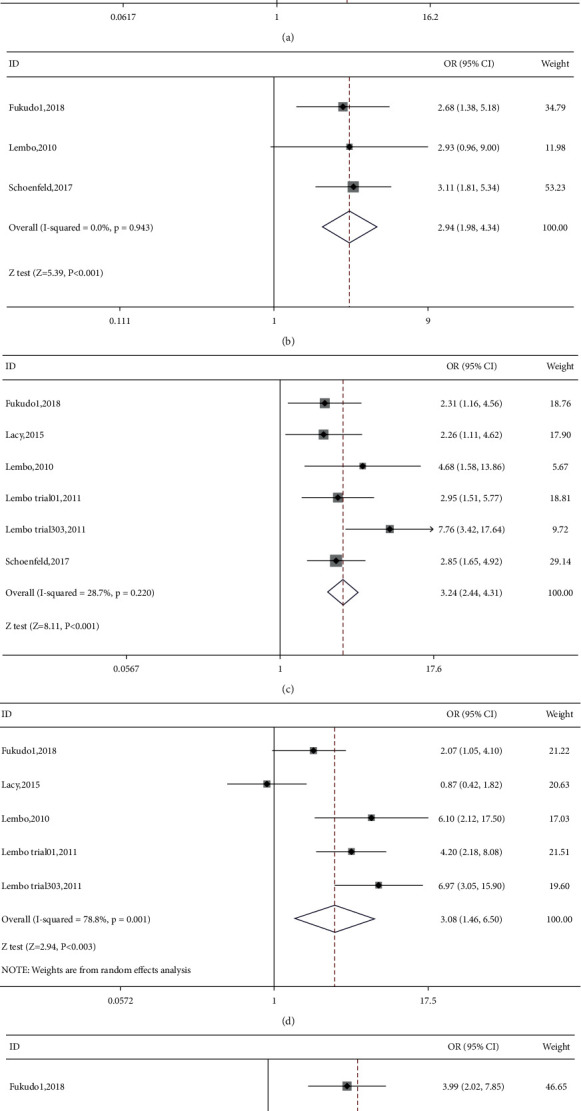
The forest plot of CSBM responder rate. (a) Linaclotide vs. placebo. (b) Extremely low dose of linaclotide vs. placebo. (c) Low dose of linaclotide vs. placebo. (d) Medium dose of linaclotide vs. placebo. (e) High dose of linaclotide vs. placebo.

Seven RCTs reported on the number of CSBM responders in the linaclotide group and placebo group. The fixed-effects model was used because no significant heterogeneity was found (*I*^2^ = 27.9% or *p*=0.216). The number of CSBM responders was significantly higher in the linaclotide group than the placebo group (OR: 3.59; 95% CI: 2.82–4.57; *p* < 0.001). Then, a stratified analysis was conducted between patients administered different doses of linaclotide and the placebo. The number of CSBM responders in the extremely low-dose group (OR: 2.94; 95% CI: 1.98–4.34; *p* < 0.001), low-dose group (OR: 3.24; 95% CI: 2.44–4.31; *p* < 0.001), medium-dose group (OR: 3.08; 95% CI: 1.46–6.50; *p*: 0.003), and high-dose group (OR: 4.79; 95% CI: 3.04–7.54; *p* < 0.001) was higher than that of the placebo, as shown in Figure 3.

#### 3.3.2. SBM Responders

Four studies conducted on 1,123 patients reported on the number of SBM responders. *I*^2^ = 45.6% or *p*=0.138 indicated that the level of heterogeneity was not significant, and the fixed-effects model was applied. Compared with the placebo group, the number of SBM responders increased significantly in the linaclotide group (OR: 2.41; 95% CI: 1.82–3.20; *p* < 0.001), as given in Table 2.

The low-dose group and the medium-dose group were further analyzed. We applied the fixed-effects model because the level of heterogeneity was not significant and found that the efficacy was comparable with the number of SBM responders between the low-dose group and medium-dose group (OR: 0.75; 95% CI: 0.50–1.12; *p*=0.156), as given in Table 2.

#### 3.3.3. Responders of the Global Assessment of Relief

A total of 3,837 patients reported the incidence of responders in the global assessment of relief. More patients reported as responders in the global assessment of relief in the linaclotide group than those in the placebo group (OR: 3.45; 95% CI: 2.28–5.22; *p* < 0.001), as given in Table 2. However, the number of responders in the global assessment of relief presented among the low-dose group vs. medium-dose group (OR: 0.95; 95% CI: 0.77–1.17; *p*=0.626) was not significant, as given in Table 2.

#### 3.3.4. Responder of Abnormal Bowel Habits Improvement

Three trials reported on responders of abnormal bowel habits improvement as an outcome. More patients in the linaclotide group presented abnormal bowel habits improvement than those in the placebo group (OR: 3.73; 95% CI: 2.59–5.36; *p* < 0.001), as given in Table 2. There was no significant improvement in abnormal bowel habits presented between the low-dose group and medium-dose group (OR: 1.29; 95% CI: 0.85–1.97; *p*=0.23), as given in Table 2.

#### 3.3.5. Responders of Abdominal Symptoms Relief

Five trials conducted on 2,307 patients reported on the number of responders of abdominal symptoms relief. More patients in the linaclotide group reported as responders of abdominal symptoms relief than those in the placebo group (OR: 2.76; 95% CI: 2.28–3.34; *p* < 0.001), as given in Table 2. However, compared to those in the medium-dose group, patients in the low-dose group presented no significant enhancement in abdominal symptoms relief (OR: 0.91; 95% CI: 0.61–1.36; *p* < 0.001), as given in Table 2.

#### 3.3.6. Adverse Effects (AEs)

Patients in the linaclotide group presented with more any AEs (RR: 1.23; 95% CI: 1.13–1.33; *p* < 0.001), gastrointestinal disorders (RR: 2.03; 95% CI: 1.34–3.09; *p*=0.001), diarrhea (RR: 3.10; 95% CI: 2.43–3.95; *p* < 0.001), and infections (RR: 1.45; 95% CI: 1.15–1.83; *p*=0.002) than those in the placebo group, as given in Table 2.

### 3.4. Bayesian Analysis for Optimal Dose of Linaclotide

**Figure 4:**
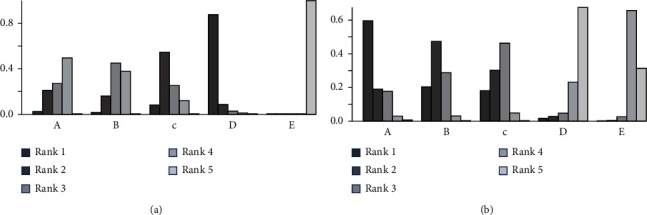
Probability rank of CSBM responders (a) and any AEs (b) by Bayesian analysis. A, B, C, D, and E were the extremely low-dose group, low-dose group, medium-dose group, high-dose group, and placebo group, respectively.

Due to no significant inconsistency, the consistency model was used to analyze the results of the CSBM responder rate and any AEs. Compared to placebo, patients with different doses of linaclotide reported more responders of CSBM. Patients with high dose of linaclotide presented the maximum rate of CSBM responders (OR: 4.94; 95% CrI: 3.22–7.79; probability rank = 0.87) indirectly compared with other dose groups, as shown in Figure 4(a). More any AEs occurred in the extremely low-dose group (RR: 1.34; 95% CrI: 1.05–1.96), low-dose group (RR: 1.26; 95% CrI: 1.06–1.62), and medium-dose group (RR: 1.23; 95% CrI: 1.03–1.67), but in the high-dose group (RR: 0.91; 95% CrI: 0.60–1.38) than those with placebo. By indirect comparison with other dose groups, extremely low dose of linaclotide obtained the highest incidence of any AE with probability rank=0.59 and high dose of linaclotide ranked the fourth (probability rank = 0.01), as shown in Figure 4(b). But by pairwise comparison of the different dose groups, no statistical significance demonstrated the outcomes of the rate of CSBM responders and the incidence of any AE, as given in Table 3.

### 3.5. Publication Bias

**Figure 5:**
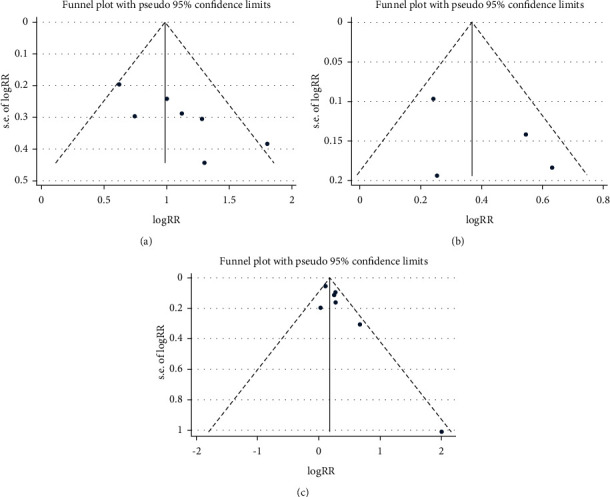
The funnel plot of outcomes between the linaclotide groups and placebo. (a) CSBM responder. (b) SBM responder. (c) Any adverse events.

A funnel plot and Begg's test were used to evaluate the publication bias, and no significant publication bias was presented for these outcomes (CSBM responders, SBM responders, and any adverse events), as shown in Figure 5.

### 3.6. Level of Evidence

The eligible studies were RCTs, and the level of evidence using GRADE instruments was high. In total, these studies provided a moderate to high level of evidence of the CSBM responder rate in Supplementary Materials (available here).

## 4. Discussion

The effects of different doses of linaclotide on CC have been assessed, resulting in confusing outcomes for clinicians. Therefore, we conducted a meta-analysis to evaluate whether a high dose of linaclotide would present more benefits for patients with CC. Up to date, this is the first meta-analysis to assess the efficacy of different doses of linaclotide. In this study, we found that the efficacy of each linaclotide dose was better than that of the placebo and was tolerable for patients with CC. Furthermore, the high dose of linaclotide was more efficacious and resulted in no more adverse events in patients with CC by indirect comparison with other dose of linaclotide.

Linaclotide, which targets GC-C receptors on the lumen of the intestinal epithelium, has been approved by the US Food and Drug Administration (FDA) for the treatment of chronic constipation (145 *μ*g daily) and IBS-C (290 *μ*g daily) in 2012 [31]. In 2017, a 72 *μ*g dose of linaclotide was approved by the FDA for treating chronic idiopathic constipation [31]. An RCT conducted at 14 clinical study centers in the United States reported that higher doses of linaclotide was associated with increased colonic transit and an increase in bowel motion frequency and consistency but reduced the straining scores of patients [32], indicating that a high dose of linaclotide may improve symptoms of patients with CC. However, a study conducted on 420 patients assessed the efficacy of linaclotide at daily doses of 75, 150, 300, or 600 *μ*g or a placebo and found no dose-dependent increase in CSBMs, SBMs, stool consistency, and straining in patients with IBS-C [33]. In our study, we found that linaclotide significantly increased the CSBM responder rate and SBM responder rate. By Bayesian analysis, it seemed the rate of CSBM responders was on the increase for higher dose of linaclotide (OR for extremely low-dose, low-dose, medium-dose, and high-dose was 3.2, 3.28, 3.66, and 4.94, respectively). But we failed to demonstrate that patients with high dose of linaclotide obtain more CSBM responders by a direct pairwise efficacy comparison between the different doses of linaclotide.

In addition, more patients administered with oral linaclotide presented the global assessment of relief (OR: 3.45), abdominal bowel habits improvement (OR: 3.73), and abdominal symptoms relief (OR: 2.76), compared with those administered the placebo. For the low dose and medium dose of linaclotide groups, the global assessment of relief responder rate was 45.70% and 46.79%, respectively, indicating that a higher dose of linaclotide provided no significant improvement in the global assessment of relief. The responder rate of abdominal bowel habits improvement and abdominal symptoms relief was 32.29% and 27.27%, and 56.83% and 58.76% between the low and medium dose of linaclotide groups, respectively, and showed no statistical significance. These results can be explained by the effect of linaclotide in reducing visceral sensitivity, as proven using rodent models of visceral pain [34]. These benefits appear to persist along with longer-term administration [21].

The most common adverse events of linaclotide are diarrhea and gastrointestinal disorders. Here, we reported the incidence of any AEs, gastrointestinal disorders, diarrhea, and infections in patients with linaclotide were all higher than those with the placebo. Most AEs were mild or moderate. Only one eligible study reported on diarrhea that resulted from the discontinuation of 0, 2.4%, and 3.2% of patients in the placebo, linaclotide 72 *μ*g group, and linaclotide 145 *μ*g group, respectively [29]. We analyze the incidence of any AEs in patients with different doses of linaclotide by the Bayesian model and found extremely low dose holds the highest incidence (RR: 1.34; 95% CrI: 1.05–1.96, probability rank = 0.59) by indirect comparison with other different doses of linaclotide. The high dose ranked the lowest (RR: 0.91; 95% CrI: 0.60–1.38, probability rank = 0.01). However, direct comparison between different doses of linaclotide showed no significant difference in increasing the incidence of any AEs, which may indicate no positive correlation between dosage and any AEs. A previous study investigated the dose of linaclotide on healthy human volunteers and found that linaclotide was well-tolerated at oral doses of up to 3000 *μ*g with no detectable change in serum levels [35]. However, one study showed that diarrhea was the only dose-dependent adverse event and was usually mild or moderate [33] which is needed to explore furtherly.

There are some limitations to this study. First, the number of eligible studies was small. Second, most studies were conducted in the United States or Japan. There was a lack of data from China and European countries. The dose of linaclotide may be different for patients of different genetic backgrounds. Third, the dose of linaclotide for different dose groups was not a single value but was a category. Fourth, efficacy parameters, such as the change in mean CSBM/SBM frequency, change in mean stool form score, and improvement of health-related quality of life, were lacking due to the unavailability of relevant data, which may change the conclusion. Finally, the definition of CC used in our study is broad and included certain secondary factors, such as medications. Therefore, there may be some discrepancies in the efficacy of different linaclotide doses between primary CC and secondary CC.

In conclusion, high dose of linaclotide could be more effective and safer for CC patients which need more trials to confirm in the future.

## Figures and Tables

**Table 1:** Baseline characteristics of the eligible studies.

Study	Country	Centers	Diagnostic criteria for CC	Total number	Female (%)	Dose of linaclotide (*μ*g)	Treatment time	Outcomes
Fukudo1, 2018	Japan	NA	Rome III	382	83.25	62.5, 125, 250, 500	2 w	CSBM responder; SBM responder; global assessment of relief; abnormal bowel habits improvement; abdominal symptoms relief
Lembo, 2010	USA	57	Rome II	310	91.86	75, 150, 300, 600	4 w	CSBM responder; SBM responder; abnormal bowel habits improvement
Fukudo2, 2018	Japan	39	Rome III	181	82.32	500	4 w	CSBM responder; SBM responder; global assessment of relief; abnormal bowel habits improvement; abdominal symptoms relief
Lacy, 2015	USA Canada	141	Rome II	483	91.5	145, 290	12 w	CSBM responder; global assessment of relief; abnormal bowel habits improvement; abdominal symptoms relief
Schoenfeld, 2017	USA	105	Rome III	1223	77	72, 145	12 w	CSBM responder; global assessment of relief
Lembo, 2011	USA Canada	212	Rome II	1276	88.64	145, 290	12 w	CSBM responder; global assessment of relief; abdominal symptoms relief
Brenner, 2020	USA	71	Rome III	252	59.9	145,290	8 w	SBM responder

**Table 2:** The summary of the secondary outcomes.

Outcomes	*Z* test	OR (95% CI)	*P*
SBM responder rate			
Linaclotide vs. placebo	6.12	2.41 (1.82, 3.20)	<0.001
Low-dose group vs. medium-dose group	1.42	0.75 (0.50, 1.12)	0.156

Responder rate of the global assessment of relief			
Linaclotide vs. placebo	5.87	3.45 (2.28, 5.22)	<0.001
Low-dose group vs. medium-dose group	0.49	0.95 (0.77, 1.17)	0.626

Responder rate of abdominal bowel habits improvements			
Linaclotide vs. placebo	7.08	3.73 (2.59, 5.36)	<0.001
Low-dose group vs. medium-dose group	1.20	1.29 (0.85, 1.97)	0.230

Responder rate of abdominal symptoms relief			
Linaclotide vs. placebo	10.42	2.76 (2.283.34)	<0.001
Low-dose group vs. medium-dose group	0.45	0.91 (0.61, 1.36)	0.65
Outcomes	*Z* test	RR (95% CI)	*P*

Adverse events between the linaclotide group and placebo			
Any adverse events	4.88	1.23 (1.13, 1.33)	<0.001
Gastrointestinal disorders	3.31	2.03 (1.34, 3.09)	0.001
Diarrhea	9.12	3.10 (2.43, 3.95)	<0.001
Infections	3.15	1.45 (1.15, 1.83)	0.002

OR, odds ratio; RR, risk ratio; CI, confident interval.

**Table 3:** Bayesian analysis of CSBM responder rate and any AEs following different dose of linaclotide.

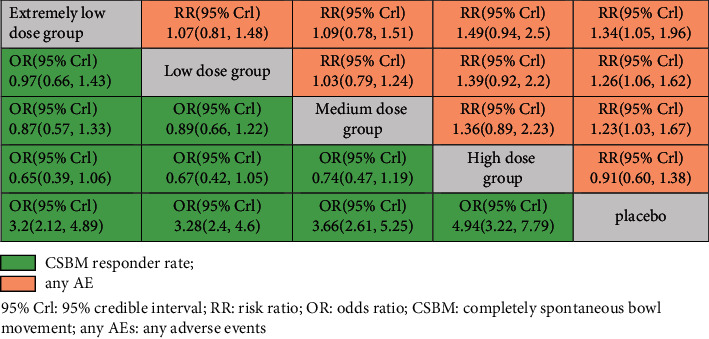

## Data Availability

The data used to support the findings of this study are available in the published articles in PubMed, the Cochrane Library, and the Embase and are included within the article.

## Supplementary Materials

Supplementary Materials Figure S1: the level of evidence of the CSBM responder rate; any AE 95% Crl, 95% credible interval; RR, risk ratio; OR, odds ratio; CSBM, completely spontaneous bowl movement; any AEs, any adverse events.
