# Supplementary material for: Comparison of the Efficacy and Safety of Different Doses of Linaclotide for Patients with Chronic Constipation: A Meta-Analysis and Bayesian Analysis
Source: Evid Based Complement Alternat Med. 2021 Oct 14;2021:9923879. doi: 10.1155/2021/9923879 (PMC8531776; doi:10.1155/2021/9923879)
Supplement: Supplementary Materials — Figure S1: the level of evidence of the CSBM responder rate; any AE 95% Crl, 95% credible interval; RR, risk ratio; OR, odds ratio; CSBM, completely spontaneous bowl movement; any AEs, any adverse events. [file 9923879.f1.pdf]

Author(s):
Question: CSBM responder compared to placebo for chronic constipation
Setting:
Bibliography: . linaclotide for chronic constipation. Cochrane□□□□□□□ [□□]□□[□□].

Certainty assessment							№ of patients		Effect		Certainty	Importance
**№ of studies**	**Study design**	**Risk of bias**	**Inconsistency**	**Indirectness**	**Imprecision**	**Other considerations**	**CSBM responder**	**placebo**	**Relative (95% CI)**	**Absolute (95% CI)**		
**CSBM responder rate□linaclotide vs placebo**												
7	randomised trials	not serious	not serious	not serious	not serious	none	573/2615 (21.9%)	89/1234 (7.2%)	**OR 3.59** (2.82 to 4.57)	**146 more per 1,000** (from 108 more to 190 more)	⨁⨁⨁⨁ HIGH	CRITICAL
**CSBM responder rate: extemely low dose group vs low dose group**												
3	randomised trials	not serious	not serious	not serious	not serious	none	102/552 (18.5%)	102/538 (19.0%)	**OR 0.93** (0.67 to 1.27)	**11 fewer per 1,000** (from 54 fewer to 39 more)	⨁⨁⨁⨁ HIGH	CRITICAL
**CSBM responder rate: extremely low dose group vs high dose group**												
2	randomised trials	not serious	not serious	not serious	not serious	none	51/141 (36.2%)	62/138 (44.9%)	**OR 0.63** (0.38 to 1.05)	**110 fewer per 1,000** (from 213 fewer to 12 more)	⨁⨁⨁⨁ HIGH	CRITICAL
**CSBM responder rate: low dose group vs medium dose group**												
5	randomised trials	not serious	not serious	not serious	not serious	none	151/710 (21.3%)	162/712 (22.8%)	**OR 0.92** (0.71 to 1.19)	**14 fewer per 1,000** (from 55 fewer to 32 more)	⨁⨁⨁⨁ HIGH	CRITICAL
**CSBM responder rate: low dose group vs high dose group**												
2	randomised trials	not serious	not serious	not serious	not serious	none	47/127 (37.0%)	62/138 (44.9%)	**OR 0.69** (0.42 to 1.15)	**89 fewer per 1,000** (from 194 fewer to 35 more)	⨁⨁⨁⨁ HIGH	CRITICAL
**CSBM responder rate: medium dose group vs high dose group**												
2	randomised trials	not serious	not serious	not serious	not serious	none	56/135 (41.5%)	57/139 (41.0%)	**OR 1.03** (0.63 to 1.68)	**7 more per 1,000** (from 106 fewer to 129 more)	⨁⨁⨁⨁ HIGH	CRITICAL
**CSBM responder rate: extremely low dose group vs placebo**												
3	randomised trials	not serious	serious	not serious	not serious	publication bias strongly suspected	106/552 (19.2%)	45/550 (8.2%)	**OR 2.94** (1.98 to 4.34)	**126 more per 1,000** (from 68 more to 197 more)	⨁⨁◯◯ LOW	CRITICAL
**CSBM responder rate: low dose group vs placebo**												
6	randomised trials	not serious	not serious	not serious	not serious	none	202/1121 (18.0%)	78/1145 (6.8%)	**OR 3.24** (2.44 to 4.31)	**123 more per 1,000** (from 83 more to 171 more)	⨁⨁⨁⨁ HIGH	CRITICAL
**CSBM responder rate: medium dose group vs placebo**												
5	randomised trials	not serious	serious	not serious	not serious	none	162/712 (22.8%)	59/644 (9.2%)	**OR 3.08** (1.46 to 6.50)	**145 more per 1,000** (from 37 more to 304 more)	⨁⨁⨁◯ MODERATE	CRITICAL
**CSBM responder rate: high dose group vs placebo**												
3	randomised trials	not serious	not serious	not serious	not serious	publication bias strongly suspected	103/230 (44.8%)	37/238 (15.5%)	**OR 4.79** (3.04 to 7.54)	**313 more per 1,000** (from 203 more to 426 more)	⨁⨁⨁◯ MODERATE	CRITICAL

CI: Confidence interval; **OR:** Odds ratio
